# Epithelial-myoepithelial parotid carcinoma after kidney transplantation

**DOI:** 10.3332/ecancer.2008.92

**Published:** 2008-08-21

**Authors:** R Horta, F Barreto, M Marques, M Rebelo, JC Reis, JM Lopes, JM Amarante

**Affiliations:** 1 Department of Plastic, Aesthetic, Reconstructive, Maxilo-Facial Surgery, and Burn Unit, Hospital de São João, Porto, Portugal; 2 Department of Pathology, Porto Medical Faculty, Hospital de São João and IPATIMUP, Porto, Portugal

## Abstract

The occurrence of a second malignant neoplasm (SMN) in patients who have been submitted to kidney transplantation is increasing and causes concern; parotid carcinoma is rarely reported after transplantation and may be related to long-term chemotherapy.

Salivary gland carcinomas displaying exclusively myoepithelial differentiation—myoepithelial carcinomas (EMC) are rare, being less than 1% of all salivary gland tumours. EMC arises most commonly in the parotid gland and usually occurs in women. Their histopathologic features, immunohistochemical profile and clinical behaviour remain controversial.

## Introduction

We here report a case of EMC occurring in a 74-year-old man, 14 years after chemotherapy (azathioprine and cyclosporine), following kidney transplantation due to chronic renal failure (hypertensive cause).

The incidence of cancers after renal transplantation is significantly higher than in the general population. It is increased by long-term immunosuppressive therapy [1] required to maintain the functional graft. The chronic use of immunosuppressive therapy in transplant recipients to prevent acute rejection increases the long-term risk of cancer. The overall incidence of *de novo* malignancies (DNM) after kidney transplantation ranges from 6% to 11%.

## Case report

The patient was followed up in the Plastic Surgery Department due to the presence of multiple malignant cutaneous neoplasms. In April 2005, he underwent excision of a scalp lesion, which was repaired with a split graft, and in January 2007, a forehead neoplasm was removed and two V-Y advancement flaps were used to cover the wound. Both lesions were epidermoid carcinomas.

In August 2007, the patient presented a left parotid swelling: fixed, solid and painless, which had been growing for two months, without signs of peripheral facial palsy.

**Figure 1:**
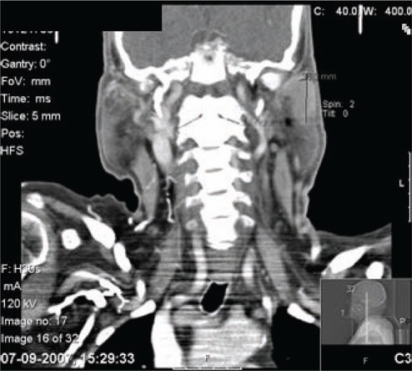
Cervical TC scan

Computed tomography scan (CTS) of the maxillofacial area showed a solid 36 × 20 mm mass, in the left parotid gland (Figure 1).

**Figure 2:**
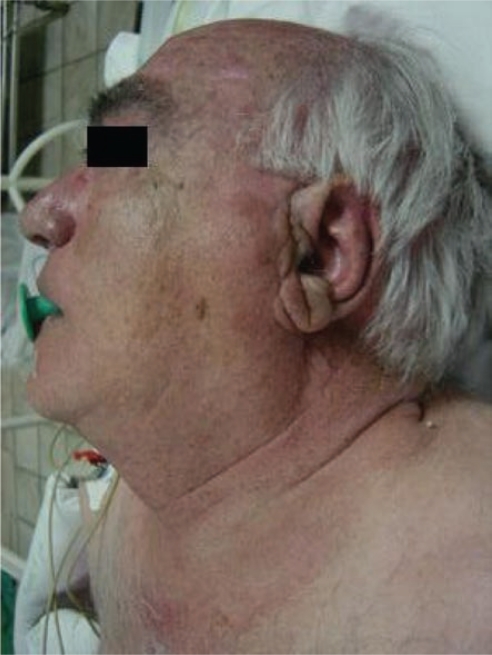
Left parotid swelling

A few small ipsilateral infra-centrimetic jugulocarotid nodes were detected, without contralateral or other salivary gland alterations (Figure 2). Fine-needle aspiration of the parotid mass was not diagnostic.

A whole body bone scintigraphy scan did not show focal or distant metastatic disease.

**Figure 3:**
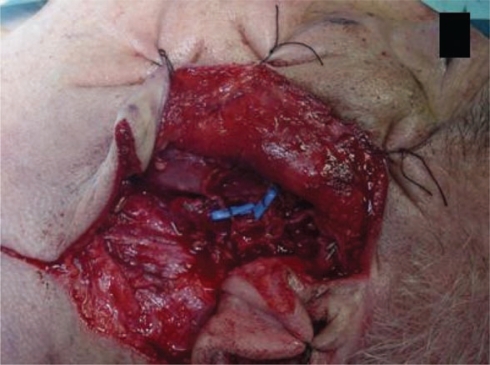
Operative view: left parotidectomy

The patient underwent a total left parothyroidectomy (Figure 3), preserving the main branches of the facial nerve except the cervical branch due to direct invasion, and to a homolateral supraomohyoid dissection.

Histologically, the mass was a carcinoma measuring 3 × 1.8 × 1.3 cm, well circumscribed and with a fibrous capsule. It was composed almost entirely of epithelioid cells with abundant clear cytoplasm and a predominantly solid pattern with hyaline extracellular matrix. The tumour cells invaded lymphatic type vessels but not the perineurial spaces. PAS staining showed positive cytoplasmic material that was diastase sensitive, that is glycogen type. One out of 36 lymph nodes (mid-jugular) showed metastatic cells. The remaining parenchyma presented with chronic inflammatory lesions.

**Figure 4:**
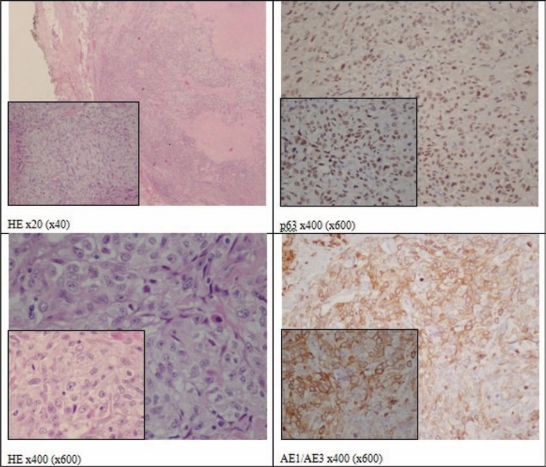
Clear cell myoepithelial carcinoma

The tumour cells expressed AE1/AE3, p63 and 34bE12 in absence of immunoreactivity for S100 protein, CD10 and CEA. These features (Figure 4) were consistent with the diagnosis of epithelial-myoepithelial carcinoma, clear-cell type, pT3, N1, M0, R2.

The patient experienced transient facial nerve paresis and underwent post-operative radiotherapy. Six months later, in follow-up, there is no sign of carcinoma recurrence.

At the present time, he is in ophthalmological follow-up because of facial nerve paresis.

## Discussion

Until recently, EMC was barely recognized. In 1985, Barnes *et al* reviewed the literature of all myoepitheliomas of the head and neck and found only three cases. Dardick *et al* [3–5] reported in 1989 and in 1995 features of the variable patterns and proposed myoepithelial carcinoma as a novel histological type of salivary gland tumours. Nevertheless, the spectrum of histological and immunohistochemical features of ECM as well as adequate treatment regimes remain controversial.

A recent study [6] summarizing new findings on salivary gland pathology has included the morphological spectrum of epithelial-myoepithelial carcinoma and variants.

1. Oncocytic variant with extensive oncocytic change in the luminal cells or in both the luminal and abluminal cells. Some of these cases may show a prominent papillary growth pattern and may mimic sebaceous type differentiation.
2. The double-clear variant with clear cell change in the abluminal myoepithelial cells, but also in luminal cells. When the epithelial component showing solid or cribriform architecture, it can be difficult to distinguish morphologically epithelial and myoepithelial cell components.

The myoepithelial component in epithelial-myoepithelial carcinoma can exhibit ‘ancient change’, that is enlarged, hyperchromatic nuclei. In contrast to de-differentiation or high-grade transformation, the chromatin is smudged. Cytological atypia is random and mitotic activity is low.

Epithelial-myoepithelial carcinoma exhibit two forms of progression:

1. overgrowth of the myoepithelial component with nuclear anaplasia. This phenomenon is also described as epithelial-myoepithelial carcinoma with myoepithelial anaplasia, when nuclear atypia in >20% of the myoepithelial cells;
2. de-differentiation to high-grade carcinoma without evidence of myoepithelial differentiation. The de-differentiated component shows high mitotic activity, and necrosis is common. They display a high recurrence rate.

Epithelial-myoepithelial carcinoma, despite the not infrequent presence of aggressive histological features, is a low-grade tumour that rarely metastasises or causes death. The most important predictive recurrence factors are surgical margin status, angiolymphatic invasion, tumour necrosis and myoepithelial anaplasia. High Ki-67 proliferative index, solid growth, size and perineural invasion all seem to significantly impact on patient survival.

In this particular case, however, we have seen an aggressive tumour with lymph-node invasion; there was no evidence of any recent skin carcinoma (previously common in this renal transplanted patient), and the histopathology revealed that the lymph-node metastasis was from the parotid carcinoma.

Due to the limited experience in the treatment of EMC, complete excision is the therapy of choice. EMC should be excised surgically with cervical lymph node dissection; post-operative radiotherapy and chemotherapy can be used but do not prevent the high rate of recurrence. Thus their use has still to be established. Metastatic sites include kidney, lung, brain, cervical lymph nodes, ribs and the scalp [7].

The NCCN guidelines [8] state that only salivary gland cancer patients with more than stage T1 disease should undergo radiotherapy (RT); this is also true for completely excised tumours with positive or close margins, intermediate or high grade, perineural or vascular invasion and lymph node metastasis. The exception is adenoid cystic carcinomas that require RT also at stage T1.

Due to their rarity, pathogenesis and consensus therapeutic guidelines are still elusive [9].

Seethala *et al* found that surgical margin status, angiolymphatic invasion, necrosis and myoepithelial anaplasia were the most significant predictors of survival [10].

An increasing number of kidney transplant patients, and the associated long-term treatment with multiple-agent chemotherapy, requires long-term follow-up with regular physical and imaging examinations. Surgeons and oncologists should be aware that second malignant neoplasm (SMN) to organs rarely susceptible to metastasis, such as the parotid gland, may occur even in patients not given radiotherapy to the head and neck region [11,12].

Immunosuppressant drugs used in organ transplantation such as cyclosporin (CiP) increase the susceptibility to infections, neoplasia and hypersensitivity reactions to drugs. The dose-dependent side effects are arterial hypertension, hypomagnesaemia, hyperuricemia, peripheral neurological symptoms, hyperlipidemia and gastric intolerance [12–15]. The main adverse cutaneous manifestations are hypertrichosis, epidermal cysts, fibrotic gingival hyperplasia and keloid folliculitis.

Regarding carcinogenesis, there is a high tendency toward the development of neoplasms, particularly lymphoid (relative risk 28 times greater than in the general population) and cutaneous lesions, related to the high doses and long-term therapy with immunosupressants and exposure to ultraviolet radiation. The cutaneous neoplasms most frequently described are epidermoid carcinoma, basal cell carcinoma, Kaposi’s sarcoma, Bowen’s disease and melanoma.

Other immunosuppressive agents also strikingly increase the risk of neoplasia, but the latency period for carcinogenesis is shorter with CiP [16]. There is a report of one case of pityriasis rubra pilaris and carcinoma of the parotid associated with CiP, retinoids and PUVA therapy [17,18].

*In vitro* studies on azathioprine have shown the development of chromosomal anomalies in pharmacological but not in therapeutic doses. Azathioprine was associated with higher incidence of cutaneous tumours, non-Hodgkin lymphomas, vulvar and cervical carcinoma, and, in association with corticosteroid treatment of kidney transplant patients, with Kaposi’s sarcoma [19]. The estimated incidence of tumours in these patients is 1–8%. It seems that there is no increase risk for other tumours in other locations, for example lung, breast, colon and prostate.

Maanziti *et al* [12] described a case of parotid carcinoma after autologous bone marrow transplantation for relapsed nephroblastoma and suggested this association with renal fossa radiation, especially when associated with multiple agent chemotherapy (doxorubicin, vincristine, actinomycin D).

We report a rare case of epithelial-myoepithelial parotid carcinoma after kidney transplantation, associated with double agent chemotherapy.

The increased risk for SMN may be dependent on a combination of genetic susceptibility, irradiation and anti-neoplastic treatment. The low risk in non-transplanted patients is probably related to the use of low doses and shorter treatments and to the absence of chronic antigenic stimulus by the transplanted organ [20].

## Figures and Tables

**Figure 5:**
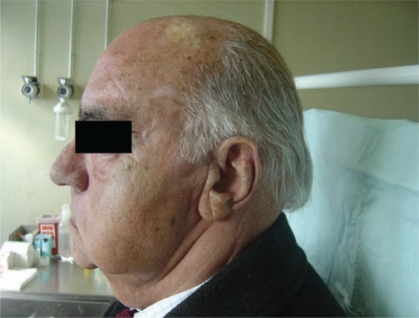
Results after 1 year

## References

[1] Ondrus D, Pribylincova V, Breza J, Buidak P (1999). The incidence of tumours in renal transplant recipients with long-term immunosupressive therapy. Int Urol Nephrol.

[2] Veroux P, Veroux M, Puliatti C, Amodeo C, Macarone M, Cappello D, Caglià P (2003). Early de novo neoplasia after renal transplantation. Tumori.

[3] Dardick I (1995). Myoepitheliomas. Definitions and diagnostic criteria. Ultrastruct Pathol.

[4] Dardick I, Cavell S, Boivin M (1989). Salivary gland myoepithelioma variants. Histological,ultrastructural, and immunocytological features. Virchows Arch A Pathol Anat Histopathol.

[5] Dardick I, Thomas MJ, van Nostrand AW (1989). Myoepithelioma—new concepts of histology and classification: a light and electron microscopic study. Ultrastruct Pathol.

[6] Cheuk W, Chan JKC (2007). Advances in salivary gland pathology. Histopathology.

[7] Savera AT, Sloman A, Huvos AG (2000). Myoepithelial carcinoma of the salivary glands: a clinicopathologic study of 25 patients. Am J Surg Pathol.

[8] (2008). National Comprehensive Cancer Network (NCCN)—Practice Guidelines in Oncology; Head and neck cancers—V.2..

[9] Tarun P, Singh K, Sharma DN, Khurana N (2004). Epithelial-myoepithelial carcinoma of the base of tongue: Pathology and management. Indian J Cancer.

[10] Seethala R, Barnes L, Hunt JL (2007). Epithelial-Myoepithelial Carcinoma: A Review of the Clinicopathologic Spectrum and Immunophenotypic Characteristics in 61 Tumors of the Salivary Glands and Upper Aerodigestive Tract. Am J Surg Pathol.

[11] Prasannan L, Pu A, Hoff P (1999). Parotid carcinoma as a second malignancy after treatment of childhood Acute Lymphoblastic Leukemia. J Pediatr Hematol Oncol.

[12] Manzitti C, Mereu P, Haupt R (2003). Parotid carcinoma after autologous bone marrow transplantation for relapsed nephroblastoma. J Pedriatic Hematol Oncol.

[13] Zanini M, Lacaz E (2001). Clinical use of Cyclosporine in Dermatology. An Bras Dermatol, Rio de Janeiro.

[14] Kazlow SD, Tripp JM, Ho VC, Lebwohl M (2005). The use of systemic immune moderators in dermatology: an update. Dermatol Clin.

[15] Ulrich C, Stockfleth E (2007). Azathioprine. UV light, and skin cancer in organ transplant patients do we have an answer?. Nephrol Dial Transplant.

[16] Singh SK, Gupta AK, Jha V, Kohli HS (2006). Treatment of oropharingeal cancer in renal transplant recipients without cessation of imunosupressive therapy. Transplant Process.

[17] Meyer P, Van Voorst PC (1989). Lack of effect of cyclosporin A in pityriasis rubra pilaris. Acta Derm Venereol.

[18] Dicken CH (1997). Treatment of classicac pityriasis rubra pilar. J Am Acad Dermatol.

[19] Dantal1 J, Pohanka E (2007). Malignancies in renal transplantation: an unmet medical need. Nephrol Dial Transplant.

[20] Gutierrez-Dalmau A, Campistol JM (2007). Immunosuppressive therapy and malignancy in organ transplant recipients: a systematic review. Drugs.

